# Preclinical Animal Models to Investigate the Role of Na_v_1.7 Ion Channels in Pain

**DOI:** 10.3390/life15040640

**Published:** 2025-04-12

**Authors:** Alvaro Yogi, Umberto Banderali, Maria J. Moreno, Marzia Martina

**Affiliations:** Human Health Therapeutics Research Centre, National Research Council Canada, Ottawa, ON K1A 0R6, Canada; umberto.banderali@nrc-cnrc.gc.ca (U.B.); maria.moreno@nrc-cnrc.gc.ca (M.J.M.); marzia.martina@nrc-cnrc.gc.ca (M.M.)

**Keywords:** Na_v_1.7, pain, neuropathic, inflammatory, pre-clinical model

## Abstract

Chronic pain is a maladaptive neurological disease that remains a major global healthcare problem. Voltage-gated sodium channels (Na_v_s) are major drivers of the excitability of sensory neurons, and the Na_v_ subtype 1.7 (Na_v_1.7) has been shown to be critical for the transmission of pain-related signaling. This is highlighted by demonstrations that gain-of-function mutations in the Na_v_1.7 gene SCN9A result in various pain pathologies, whereas loss-of-function mutations cause complete insensitivity to pain. A substantial body of evidence demonstrates that chronic neuropathy and inflammation result in an upregulation of Na_v_1.7, suggesting that this channel contributes to pain transmission and sensation. As such, Na_v_1.7 is an attractive human-validated target for the treatment of pain. Nonetheless, a lack of subtype selectivity, insufficient efficacy, and adverse reactions are some of the issues that have hindered Na_v_1.7-targeted drug development. This review summarizes the pain behavior profiles mediated by Na_v_1.7 reported in multiple preclinical models, outlining the current knowledge of the biophysical, physiological, and distribution properties required for a Na_v_1.7 inhibitor to produce analgesia.

## 1. Introduction

Pain is a leading global healthcare problem, with chronic pain, in particular, being a major concern, affecting 1 in 10 adults annually. Treatments for chronic pain usually involve a variety of approaches, including, but not limited to, pharmacological interventions, physical exercise or therapy, and psychological support [1]. Although many types of pain are well managed with current medications, the development of new pharmacological agents remains an urgent need. For some chronic pain conditions, such as neuropathic pain, cancer, or chemotherapy-induced pain, the available drugs have partial or limited efficacy and, in some cases, can provoke addiction and abuse [2,3]. The widespread use of opioid-derived painkillers is one of the primary causes of the worldwide opioid crisis, which causes thousands of deaths in many countries and represents a major health and social challenge in modern society [4].

**Figure 1:**
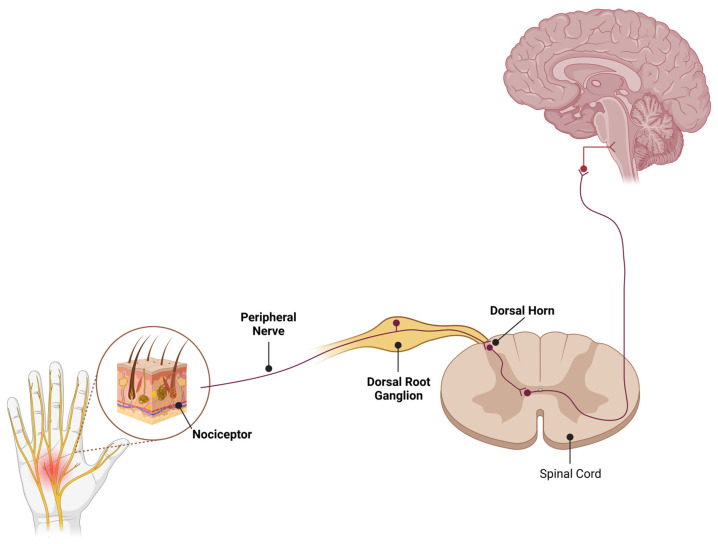
Schematic diagram of pain pathways where high Na_v_1.7 expression has been reported. Na_v_1.7 display robust expression in peripheral sensory neurons of all sizes. In addition, expression of Na_v_1.7 is detected in both central and peripheral projections of DRG neurons along with the central terminals of sensory neurons in the laminae I and II of the dorsal horn. Illustrations created in BioRender (www.biorender.com).

The sensation of pain originates in the peripheral nervous system (PNS) through the activation of nociceptors, which are remarkably heterogeneous in nature, encompassing various types of receptors and ion channels [5]. Among them, transient receptor potential (TRP) channels are widely expressed in sensory neurons and mediate the transduction of noxious (thermal, electrical, and mechanical) stimuli into electric impulses. Pain-associated TRP channels include members of the subfamilies ankyrin (A), vanilloid (V), melastatin (M), and canonical (C), and their roles in physiological and pathological conditions, including pain, have been expertly reviewed [6]. The nociceptive signals travel from the sensory neurons of the dorsal root ganglia (DRG) to neurons in the dorsal horn of the spinal cord, where they are finally transmitted to the brain to be processed [7]. The transmission of pain signals is mediated by the activity of ion channels present in the membranes of the neurons responsible for the generation and propagation of the action potentials. In particular, the voltage-gated sodium channels (Na_v_s) are fundamental players in this process, triggering and modulating the amplitude, duration, and frequency of action potentials [8] (Figure 1).

Na_v_s are involved in the control of many fundamental physiological processes in various tissues of the human body. The Na_v_ family 1 consists of nine members, each represented by pore-forming α subunits, which, in mammals, are numerically named from Na_v_1.1 to 1.9 and are encoded by the genes SCN1A, SCN2A, SCN3A, SCN4A, SCN5A, SCN8A, SCN9A, SCN10A, and SCN11A, respectively [9]. Although these isoforms share a common overall structural motif, each Na_v_ subtype has a different function and exhibits different expression profiles [10]. Na_v_1.1, Na_v_1.2, Na_v_1.3, and Na_v_1.6 are mainly expressed in the central nervous system (CNS) [11]; Na_v_1.7, Na_v_1.8, and Na_v_1.9 are present in the PNS; Na_v_1.4 is the skeletal muscle sodium channel; and Na_v_1.5 is the predominant cardiac myocyte channel [12,13,14].

For decades, the pharmaceutical industry has pursued drugs modulating Na_v_ channel activity in the search for effective painkillers. Unfortunately, these attempts have often resulted in drugs with an insufficient therapeutic index (i.e., the efficacy–safety ratio). The ideal analgesic should be able to reduce the pain sensation at the PNS level without having adverse effects on the CNS and without interfering with other organs’ functionality. Na_v_1.7, Na_v_1.8, and Na_v_1.9, expressed in nociceptors, are among the most studied targets for pain treatment [15,16]. Na_v_1.7, in particular, is responsible for the initiation of the action potential in pain responses, and, due to this critical role, its dysfunction is associated with several pain related disorders. Genetic studies have shown that mutations in the SCN9A gene, which encodes the Na_v_1.7 protein, result in familial pain disorders [17]. Mutations in the SCN9A gene can be categorized as gain-of-function mutations (increased Na_v_1.7 activity), causing paroxysmal extreme pain disorder (PEPD) and erythromelalgia (EM); or loss-of-function mutations (reduced Na_v_1.7 activity), causing congenital insensitivity to pain (CIP) [18]. An important characteristic of the patients suffering from CIP is that even in the total absence of Na_v_1.7 activity, they do not exhibit any cognitive, cardiac, motor, or sensory deficits, supporting Na_v_1.7 as a valid—and indeed attractive—target for the development of drugs against pain. Interestingly, rodent models of neuropathic pain showed increased Na_v_1.7 activity, further supporting the importance of Na_v_1.7 as a pain target [19,20,21]. These findings have fueled a race to develop a myriad of molecules targeting Na_v_1.7 for pain applications.

## 2. Targeting Na_v_1.7 for Pain Applications

**Figure 2:**
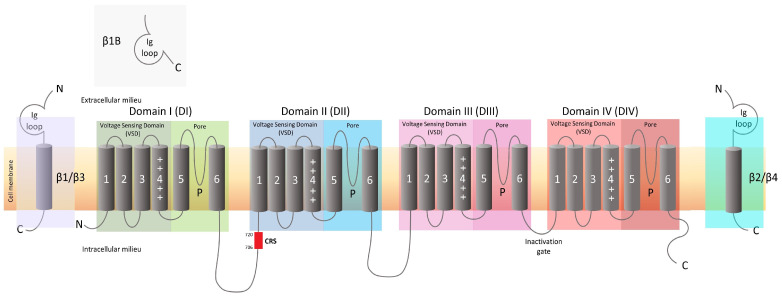
Schematic of the α and β subunits of the Na_v_ channel. The four domains of the α subunit of Na_v_ channels are depicted as follows: Domain I (DI, green); Domain II (II, blue); Domain III (DIII, violet); and Domain IV (DIV, red). Each domain contains six transmembrane helices (1–6). Helices 5 and 6 form the pore region of the channel; the positively (+) charged helix 4 as well as helices 1–3 form the voltage-sensing domain (VSD). The inactivation gate is located in the intracellular loop connecting helix 6 of DIII to helix 1 of DIV. The P-loop between helices 5 and 6 serves as a selectivity filter. In the intracellular loop between DI and DII, the collapsin response mediator protein 2 (CRMP-2) regulatory sequence (CRS; red rectangle) is shown. The β1 (purple), β2 (azure), β3 (purple), and β4 (azure) consist of an extracellular immunoglobulin loop (Ig loop), a transmembrane helix, and an intracellular C domain. The β1B (grey) is soluble and lacks the transmembrane segment with the Ig loop directly connected to the C terminal.

Na_v_s are protein complexes constituted by one α subunit and two auxiliary β subunits. The α subunit is the ion-transporting unit of the complex, consisting of ∼2000 amino acids. Its structure is composed of four similar domains (DI to DIV), each containing six transmembrane-spanning helices with a pore region located between helices 5 and 6 of each domain [22]. The helices from 1 to 4 constitute the voltage-sensing domain (VSD), which regulates the opening of the channel during membrane depolarization. The auxiliary β subunits have multiple functions, including the modulation of the channel’s kinetic and gating activity, as well as the trafficking of the protein complex to the plasma membrane (Figure 2).

The first class of small molecules targeting Na_v_s, developed in the first half of the last century (e.g., novocaine and lidocaine), had low specificity. As a result, their analgesic effect could only be achieved via topical application to avoid dangerous side effects. Today this type of analgesic is commonly used as local anesthetic during dental procedures.

In the past decade, small molecules highly specific for Na_v_1.7 have been developed; however, they had little analgesic effect in the human and failed early phases of clinical trials. For example, Pfizer’s PF-04856264, despite its high affinity and potency for Na_v_1.7, had no effect in reducing pain in formalin-, carrageenan-, or complete Freund’s adjuvant (CFA)-induced acute pain models in mice [23]. Although it was able to partially reduce the pain behaviors induced by the α scorpion toxin OD1, a Na_v_1.7 channel activator, and in surgery-induced mechanical allodynia when injected locally, no effect was observed when administered systemically [24,25]. Subsequently, Pfizer advanced an optimized version of PF-04856264, named PF-05089771, into clinical trials, where it showed no significant improvement versus placebo or the control drug [26,27]. The disappointing analgesic performance of the small molecules targeting Na_v_1.7 in the clinic has been proposed to be due to their high binding to plasma proteins, thus leading to low concentrations at the target-binding site [27,28]. Other high-affinity Na_v_1.7 inhibitors have undergone Phase I trials, including AstraZeneca’s AZD-3161 (NCT01240148), Genentech’s GDC-0276 (NCT02856152), and Sumitomo Dainippon Pharma’s DSP-2230. However, they have since been discontinued from their respective development pipelines [29].

Several laboratories are actively trying to develop novel analgesics by altering the chemical structure of molecules such as aryl sulfonamides, acyl sulfonamides, or natural toxins (e.g., saxitoxin and ProTX) that have shown inhibitory effects on Na_v_1.7. These efforts aim to improve their affinity, potency, selectivity, stability, and clearance rate [30,31,32]. Using chemical and docking studies of the Na_v_s binding sites for neurotoxins and other drugs, Na_v_s inhibitors are being designed to interact with the canonical binding sites of Na_v_1.7 [28]. Two of these binding sites are located at the level of the selectivity filter and are accessible independently of the kinetic state of the channel. One of such binding sites is located in the central pore cavity (formed by helices 5 and 6) and is accessible either from the cytoplasm or, more often, through interdomain fenestrations. This is the site where the local anesthetics (e.g., lidocaine) bind. The other binding site, facing the extracellular side of the pore and lining the pore helices, binds marine toxins such as tetrodotoxin or saxitoxin. Molecules binding to these two sites block the channel by preventing the flux of ions inside the cell and locking it in a non-conductive state. A third binding site is located in the VSD of DII at the extracellular junction of the helices 1–2 and 3–4, and it is accessible only when the channel is in its resting state. A class of spider toxins (e.g., ProTX-II) binds to this site, preventing the movement of the VSD towards its “active” position upon membrane depolarization, locking the channel in a closed conformation. A fourth site is located in the helices 1–2 and 3–4 regions of the DIV VSD. Sulfonamide-based compounds preferably bind to this site, which is only available in the active conformation of the channel with the membrane depolarized. Upon binding, blockers trap the channel in an inactivated state [28].

Despite showing great promise during the discovery phase, numerous analgesic candidates targeting not only Na_v_1.7 but also other novel targets have failed in their translation to the clinic. As such, currently, no Na_v_1.7 small molecule inhibitor has advanced to Phase III clinical trials [26,27,33]. Reasonings for this failure include poor pharmacokinetics, suboptimal clinical trial design, toxicity issues, and/or poor preclinical modeling [34]. For instance, it is now clear that high affinity for Na_v_1.7 does not necessarily translate in effective pain reduction, as demonstrated by PF-04856264. This highlights the importance of testing drugs on various animal pain models that can discriminate which types of pain these new entities may effectively target. Moreover, the identification of pain states that are independent of Na_v_1.7 activity further highlights the importance of choosing appropriate models [35,36]. Taken together, these lessons have paved the way for the discovery of a large number of new or “improved” molecules with the potential to be developed into new clinically relevant analgesics. Nonetheless, in vivo efficacy testing in multiple models are likely required to determine the specific pain types these molecules can address. The goal of this review is to provide an overview of the role of Na_v_1.7 in mediating pain responses in the most commonly used preclinical pain models. We aim to provide valuable insights regarding the use of these models for screening the activity of potential new therapies targeting Na_v_1.7. A fundamental understanding of the mechanisms involved in pain transmission is imperative for appropriately examining the therapeutic potential of Na_v_1.7 channel inhibitors for analgesic applications.

## 3. Preclinical Discovery of Analgesic Compounds

### 3.1. Animal Models of Pain

**Figure 3:**
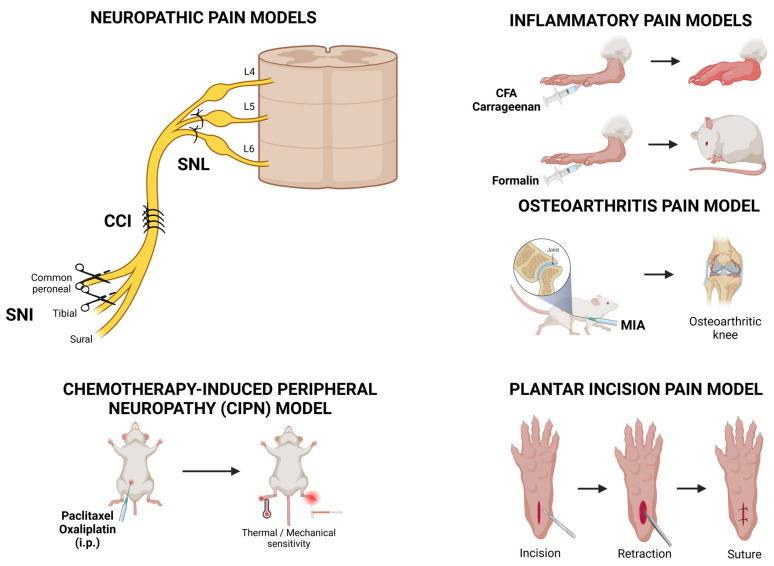
Overview of various pre-clinical pain models used in Na_v_1.7 research. Among the neuropathic pain models, the ligation of the distal dorsal root ganglion (DRG) is used to induce the spinal nerve ligation (SNL) model. The chronic constriction injury (CCI) model is established by ligating the main trunk of the sciatic nerve, whereas the spared nerve injury (SNI) model ligates the tibial nerve and the common peroneal nerve, leaving the sural nerve intact. Chemotherapy-induced peripheral neuropathy (CIPN) models are obtained following the intraperitoneal administration of chemotherapeutic drugs, resulting in the development of thermal and mechanical sensitivity. Inflammatory pain models involve the intraplantar injection of complete Freund’s adjuvant (CFA), carrageenan, or formalin. Osteoarthritis pain models are obtained with an injection of monoiodoacetate (MIA) to the knee joint, leading to pathological changes that resemble human osteoarthritis. Finally, the plantar incision pain model has been developed as a preclinical tool to identify the molecular, cellular, and physiological mechanisms that underlie postoperative pain. Illustrations created in BioRender.

Animal models are essential for understanding disease mechanisms and testing novel therapies, particularly in pain research, due to the practical and ethical concerns associated with human experimentation. Overall, analgesic drugs are effective in animal models, indicating that similar nociceptive machinery is present. Pain models can be broadly categorized into inflammatory (e.g., CFA, carrageenan, and formalin test), neuropathic (e.g., spinal nerve ligation (SNL), chronic constriction injury (CCI), spared nerve injury (SNI)), arthritic (e.g., monoiodoacetate (MIA)-induced arthritis model), chemotherapy-induced peripheral neuropathy (CIPN), and postoperative pain models (e.g., plantar incision model) (Figure 3).

#### 3.1.1. Complete Freund’s Adjuvant (CFA) and Carrageenan-Induced Pain Models

Inflammatory pain models rely on the administration of a chemical inflammogen or causing tissue damage via mechanical or thermal means. CFA and carrageenan are two of the most commonly used agents [37,38]. CFA is a suspension of heat-killed *Mycobacterium butyricum* or *Mycobacterium tuberculosum*, whereas the carrageenan are sulfated polysaccharides extracted from seaweed. Injection of these compounds into the paw of rodents induces edema and thermal and mechanical hyperalgesia. This pain hypersensitivity develops within 30 min post injection, peaks between 2 h and 1 day, and can persist for at least 7 days [39,40]. Inflammatory pain results from the sensitization of primary afferent nociceptive fibers innervating the inflamed tissue. This phenomenon is mediated by a complex series of events, involving the activation of multiple signal transduction pathways and the modulation of neuronal function and activity, resulting in lowered activation thresholds and increase evoked afferent activity [41,42]. In particular, the modulation of Na_v_1.7 channels has been shown to have a prominent role in inflammatory pain.

#### 3.1.2. Formalin Test

The formalin test has been widely used as a valuable tool for rapidly screening the efficacy of potential analgesic drugs [43,44]. Intraplantar injection of formalin elicits a spontaneous biphasic nocifensive behavior that involves the licking, flinching, and biting of the injected paw. The phase 1 (acute phase) is characterized by intense pain believed to result from the direct chemical stimulation of nociceptors [45]. This is followed by an interphase, a period of reduced pain, and finally by a final phase, during which pain resumes (second phase). This second phase is thought to reflect ongoing inflammation and sensitization of the dorsal horn in the spinal cord, although this view was recently contested by Hoffmann and collaborators [46]. A great advantage of the formalin test is that it is relatively simple to perform as it does not require complex surgical procedures or specialized equipment. Furthermore, positive behavioral responses are obvious and easily quantifiable.

#### 3.1.3. Chronic Constriction Injury (CCI) Model

The CCI model is a widely used model of neuropathic pain that mimics the lesions of nerve fibers located at the surface of the peripheral nerves and reproduces the main sensory symptoms associated with neuropathic pain in humans [47]. In this model, ligatures loosely tied around the sciatic nerve induce long-lasting chemical and thermal hypersensitivity, cold and mechanical allodynia, and spontaneous pain [48,49].

#### 3.1.4. Spared Nerve Injury (SNI) Model

The SNI involves ligating and severing two terminal branches of the sciatic nerve, the common peroneal and tibial nerves, while leaving the sural nerve intact [50]. This procedure leads to the development of mechanical and thermal hypersensitivity in the area innervated by the sural nerve only on the ipsilateral side, but not the contralateral side. Mechanical hypersensitivity is observed within 3 days post-surgery, peaks around 14 days post-surgery, and lasts for at least 4 weeks [51,52]. Originally described in rats, the SNI model has since been adapted and validated in mice [53]. One major implication of performing SNI studies in mice versus rats is the origin of sciatic never fibers. In rats, the majority of sciatic nerve fibers originate from the L4 and L5 DRG [54]. However, in mice, the functional and anatomical equivalents of the rat L4 and L5 DRG are instead the L3 and L4 DRG [55]. This distinction was further demonstrated in the SNI model, where ATF3 immunoreactivity, a marker of axotomized neurons, was significantly increased in the L3 and L4 DRG 7 days after surgery. In contrast, the percentage of ATF3-positivity in the L5 DRG was similar to that of sham animals [56].

#### 3.1.5. Spinal Nerve Ligation (SNL) Model

The SNL, which involves the ligation and transection of the L5 spinal nerve, is one of the most widely used models of neuropathic pain in rodents [57]. This procedure results in spontaneous ectopic discharges in afferent A-fibers (Aβ and Aδ), which are believed to be a key driver of the abnormal sensations observed in this model [58]. The spinal nerve transection of the fifth lumbar segment model is a slight modification of that described originally by Kim and Chung [57]. In this model, two ligatures, approximately 5 mm apart, are placed in the L5 spinal nerve, followed by a transection between the ligatures [59]. In both models, animals develop long-lasting spontaneous pain, hyperalgesia, and allodynia in the ipsilateral hind paw within a few days after surgery [57,59,60].

#### 3.1.6. Spinal Cord Injury (SCI) Models

In clinical settings, SCI is an exceptionally heterogeneous condition, and as such, several animal models have been developed with variable locations, extents, and means to induce injury [61]. Blunt direct trauma by contusion that mimics clinical traumatic injuries, ischemic lesions, and complete or partial spinal cord transection are some of the methods of inducing SCI in preclinical studies, and they are associated with the development of mechanical allodynia and thermal hyperalgesia following model induction [62,63,64,65,66].

#### 3.1.7. Chemotherapy-Induced Peripheral Neuropathy (CIPN) Models

Paclitaxel (Taxol^®^, Princeton, NJ, USA) is a chemotherapeutic agent commonly used in the treatment of proliferative breast, ovary, and lung cancers [67]. It acts by stabilizing microtubule scaffolding within treated cells, thus blocking the mitotic spindle dynamics required for cell division. This results in cell cycle arrest and apoptosis of the rapidly dividing cancer cells [68]. However, known side effects of paclitaxel treatment are its pathological effects on sensory neurons of the peripheral nervous system, a condition known as CIPN [69]. Clinically, CIPN manifests as chronic pain, often localized in the hands and feet [70]. In the rodent model, the chemotherapy agent is typically administered through intravenous or intraperitoneal injections four times, separately, at doses that range from 2 to 8 mg/kg, administered every other day, which is thought to mimic one cycle of chemotherapy treatment in humans [71]. The pathophysiological processes induced by paclitaxel include inflammation, oxidative stress, loss of primarily epidermal nerve fibers, and alterations to the mitochondrial function and excitability of peripheral neurons.

#### 3.1.8. Plantar Incision Model of Postoperative Pain

Postoperative pain is the most common form of acute pain, and a major concern is that following this phase, a significant number of patients will develop chronic pain that will severely impact their quality of life [72]. Rat and mouse models of acute incisional pain have been developed as preclinical tools to identify the molecular, cellular, and physiological mechanisms that underlie postoperative pain [73,74,75]. The hind paw incision model in rodents causes reversible edema around the wound site and is characterized by non-evoked pain behavior, as well as mechanical and heat hyperalgesia, that lasts for several days [74]. It is an established and reliable model of postoperative pain that greatly simulates the clinical features observed in patients. Indeed, the main advantage of the plantar incision model is that the pain following the skin and muscle incision more closely mimics the inflammatory pain process in patients than other models such as the CFA- or carrageenan-induced models.

#### 3.1.9. Osteoarthritis (OA) Models

Osteoarthritis is a disabling, degenerative disorder marked by progressive joint failure, which is associated with cartilage breakdown and a loss of the extracellular matrix, which, under physiological conditions, provides the compressive resilience essential for joint function [76]. Surgical and chemical induction in mice are the two most common preclinical models of OA, and, similar to the pathogenesis in humans, both methods exhibit articular cartilage erosion or loss and OA-related pain [77]. Injection of sodium iodoacetate (MIA model) or CFA into the knee joint are the two most commonly used models of OA and are associated with the progressive degradation of the cartilage and subchondral bone lesions in the knee [78,79,80]. At the molecular level, these models involve the up-regulation of the neuronal damage marker cAMP-dependent transcription factor ATF-3 in the peripheral nerves that innervate the knee joint, a reduction in intra-epidermal nerve fiber density, and alterations in neuroimmune cells within the spinal cord [81,82]. The MIA model, in particular, presents robust mechanical and cold allodynia, along with a significant decrease in hind limb weight-bearing on the ipsilateral side two weeks post-induction [80,83,84].

### 3.2. Mechanical and Thermal Evoked Tests

Pain tests are classified based on nociception modalities that can be of mechanical (i.e., von Frey, mechanical conflict) or thermal (i.e., Hargreaves assay, hot and cold plate, temperature place preference) nature [85,86]. Mechanical allodynia refers to the perception of pain in response to non-painful stimuli such as light touch or pressure, and it is a crucial indicator of neuropathic pain development. One of the most widely used methods of assessing mechanical allodynia is the von Frey filament test, which involves applying a series of calibrated filaments to the animal’s paw to determine the pain sensitivity threshold, i.e., the point at which the animal responds to the mechanical stimulus [87]. Traditionally, standard Von Frey hairs are employed for this test; however, more recently, electronic von Frey algometers have emerged as the preferred choice due to their higher reliability, precise control over the applied force, and consistent rate of stimulus application, which allows for standardized testing conditions across experiments and consistency [88].

Thermal nociception is another critical method for assessing pain in preclinical research. This test involves applying a radiant heat source to the animal’s paw, triggering a withdrawal reflex. The latency period between the heat stimulus application and the withdrawal response is then measured, providing a reliable metric for heat sensitivity. This test demonstrates a remarkable ability to differentiate between diverse analgesic agents and is highly reliable in evaluating heat hyperalgesia in animal models [37]. These behavioral tests allow researchers to quantitatively evaluate abnormal pain sensations that develop as complications in pre-clinical pain models, and they are routinely used to investigate the potential analgesic effects of a myriad of compounds.

Experimental models of pain sensitivity include testing threshold responses to high-intensity stimuli such as heat. Acute thermal pain is commonly assessed by the hot-plate and tail-flick tests, where the latency of the animal’s response to the stimulus is recorded as the dependent variable [89]. In the hot-plate test, animals are injected with test compounds or controls before being placed on a hot plate. The latency to respond to the heat stimulus is measured as the time it takes for the animal to react by jumping or licking the affected paw. The hot-plate test is a rapid, inexpensive, reliable, and reproducible assessment that measures the supraspinal responses to nociception without causing inflammation [89]. The temperature is typically set at 52 or 55 °C, which allows for the observing of baseline response latencies of approximately 10 s for paw licking or other observable responses. In the past decade, a modified hot-plate test has been developed, where the temperature is gradually increased from non-noxious to noxious levels. Such dynamic hot plates provide precise control over temperature ramps, allowing for the differentiation between thermal allodynia and thermal hyperalgesia [90].

The tail-flick test is widely used to assess acute nociception, involving the application of a high-intensity thermal stimulus to the tail of a mouse or a rat. The time from onset of stimulation to the animal’s rapid tail flick or withdrawal from the heat source is recorded as the primary outcome measure [89]. One advantage of this test is its reduced dependency on the animals’ motor coordination compared to the hot-plate test. Unlike the hot-plate test, where the endpoint consists of a complex behavior such as paw licking or jumping, this test induces a simple, spinally mediated flexion reflex response. This is an important distinction as it highlights the different levels of nociceptive processing assessed by the two tests. The hot-plate test primarily evaluates supraspinal responses, whereas the tail-flick test measures spinal nociceptive reflexes.

Tamoxifen-inducible Na_v_1.7 knockout (KO) mice (Na_v_1.7 cKO) display decreased SCN9A mRNA expression in tissues that normally express Na_v_1.7, including trigeminal ganglia and DRG neurons and sympathetic ganglia. These Na_v_1.7 conditional KO mice, as well as mice with global Na_v_1.7 KO (Na_v_1.7 gKO), show prolonged response latencies to noxious heat in the hot-plate test [91,92]. These findings are further corroborated by studies using animals with Na_v_1.7 deletions in specific subsets of neuronal populations. For instance, transgenic Na_v_1.7^Wnt1^ mice, generated by using the Wnt1 promoter to drive Cre expression in neuronal crest derivatives (including autonomic and sensory neurons), demonstrated attenuated responses in the hot-plate test [93]. Similarly, the use of Advillin (Na_v_1.7^Advill^) and Wnt1 (Na_v_1.7^Wnt1^) promoters for the generation of conditional Na_v_1.7 KO strains allowed for the specific deletion of the channel in DRG and sensory and sympathetic neurons, respectively [35]. The same study also indicates that Na_v_1.7 KO animals with specific deletions in the DRG (Na_v_1.7^Advill^) or in sensory neurons driven by the Na_v_1.8 promoter (Na_v_1.7 ^Nav1.8^) have normal hotplate pain behavior [93]. Interestingly, performing similar studies using a heat ramp paradigm suggests that different heat stimulus intensities require distinct neuronal subpopulations. When temperature is increased at rate of 0.6 °C/s, a significant increase in response threshold is observed not only for Na_v_1.7^Wnt1^ mice but also Na_v_1.7^Nav1.8^, Na_v_1.7^Advill^ when compared to littermate controls [94]. However, when a heat ramp of 2 °C/s was applied to the plantar surface of the hind paw, only Na_v_1.7^Advill^ and Na_v_1.7^Wnt1^ displayed increased withdrawal latency. The requirement of Na_v_1.7 channels in Na_v_1.8-positive sensory neurons under certain conditions was further validated by Hockley and collaborators, who demonstrated that Na_v_1.7^Nav1.8^ mice displayed an increased latency and thermal threshold in the ramping hot-plate test [95].

Cold sensitivity can be determined by a variety of behavioral methods. One of the simplest methods is the acetone evaporation test, which involves dabbing or spraying acetone on the mouse paw and measuring the time the mouse spends flicking the paw [96]. The acetone evaporation produces a cold stimulus that is typically non-nociceptive in naïve animals but induces cold allodynia in certain pain models [96]. In addition, cold plates may also be used to assess nociceptive responses that include paw withdrawal latency at a specific temperature, the number of flinches within a defined timeframe, or the aversive reaction to a cooling ramp that determines the cold response threshold [97,98]. This test is widely used in both inflammatory and nerve-injury models, where it has been demonstrated that the withdrawal threshold for temperatures of 15 °C, a non-painful temperature in naïve rats, is significantly reduced [99].

Cold-plate tests indicate that behavioral responses to noxious cold are Na_v_1.7-independent. This is based on observations that deletion of Na_v_1.7 in the DRG, Na_v_1.8-positive sensory neurons or in autonomic and sensory neurons has no effect on aversion to extreme cold [93]. These results are further corroborated by evidence that neither Na_v_1.7^Nav1.8^ mice nor the application of the selective Na_v_1.7 antagonist PF-5198007 (another variation of PF-04856264) affects cold-evoked afferent firing [95]. Interestingly, responses to noxious cooling triggered by acetone application appear to depend on Na_v_1.7 expression in Na_v_1.8-negative sensory neurons [93]. This is based on observations that the behavioral responses in Na_v_1.7^Nav1.8^ KO mice were not significantly different from littermate controls, whereas deletion of Na_v_1.7 in the DRG, or in autonomic and sensory neurons in the Na_v_1.7^Advill^ and Na_v_1.7^Wnt1^ KO mice, respectively, had no effect on the behavioral responses induced by acetone [93].

Of note, the use of behavioral tests, such as responses to von Frey filaments or temperature gradients, rely on reflexive responses to painful stimuli, which are not necessarily representative of the clinical condition and potentially limit the translation of their findings. More recently, alternative options to reflective response testing have emerged. These include the assessment of facial expression changes using the mouse grimace scale, condition and temperature place preference tests, and evaluations of balance, coordination, and walking gait [100]. While stimulus-free pain response quantification more closely mimics clinical settings, these tests have not been widely adopted due to challenges that range from cost to difficulty of implementation and require further refinement and validation in animal models. Nonetheless, these methods offer a new avenue for objective preclinical pain assessment that does not rely on the application of noxious stimuli and that better resembles the symptomatology of pain observed in clinical settings.

## 4. Na_v_1.7-Mediated Reversal of Hyperalgesia in CFA and Carrageenan-Induced Pain Models

Following CFA injection, c-fiber nociceptors are sensitized, leading to increased spontaneous firing activity and pain behaviors [101]. This effect can be blocked by compounds targeting Na_v_1.7. Interestingly, while evoked responses of C-fiber nociceptors were reduced but not completely abolished, a dose-depended reversal of thermal hyperalgesia was observed in the CFA model [102]. In addition, the expression of Na_v_1.7 has been shown to be significantly increased in the DRG neurons following the administration of complete CFA or carrageenan [40,103,104]. This rapid and prolonged upregulation correlates with the development and maintenance of both thermal and mechanical hyperalgesia mediated by cyclooxygenase and the Akt-dependent pathways [40,104]. In the carrageenan inflammatory model, the development of thermal hyperalgesia was absent in Na_v_1.7 nociceptor-specific KO mice [105]. Similarly, hyperalgesia, induced by the intraplantar injection of CFA, is absent in global Na_v_1.7 KO (Na_v_1.7 gKO) mice, as well as in the Na_v_1.7 nociceptor-specific KO (Nav1.7R^−/−^) mice [92,105]. Of notice, in a tamoxifen inducible KO (Na_v_1.7 cKO) mouse model that enabled the genetic deletion of Na_v_1.7 in adult mice, thermal but not mechanical hypersensitivity was abolished [91]. Importantly, in none of these models were there differences in edema formation when compared to wild-type controls. The role of Na_v_1.7 in inflammatory pain is further highlighted by the findings that knockdown of Na_v_1.7 in the DRG blunts thermal hyperalgesia induced by CFA injection [106]. In addition, repression of Na_v_1.7 expression in the DRG using epigenome engineering platforms reduced heat hyperalgesia in the carrageenan inflammatory pain model, further supporting the critical role of Na_v_1.7 in hypersensitivity to heat following inflammation [107].

Given the simplicity of inducing injury and the reliability of the method, it is not surprising that numerous studies have demonstrated the reversal of hyperalgesia by multiple agents targeting Na_v_1.7. From localized intraplantar or intrathecal injections to systemic administration by oral, intraperitoneal, or intravenous routes, Na_v_1.7 inhibition has shown variable efficacy in alleviating CFA-induced thermal and mechanical hyperalgesia in rodents [108,109,110]. The route of administration, the pharmacokinetics, and the biodistribution of test compounds are important factors to consider when testing the efficacy of Na_v_1.7 inhibitors. For instance, whereas intrathecal administration of GpTx-1-71 inhibited CFA-induced hyperalgesia in rats with an ED50 value of 6.90 pmol, intraplantar injection of the same compound had an ED50 of 6.66 nmol, which is roughly 1000-fold less potent [109]. This further highlights a significant challenge in the development of Na_v_1.7 inhibitors. Despite the discovery of many compounds with high potency in vitro, many fail to achieve sufficient target engagement in vivo [111].

## 5. Role of Na_v_1.7 in Mediating Pain Behaviors in the Formalin Test

Formalin administration has been shown to activate mitogen-activated protein kinase (MAPK) signaling pathways through interleukin-33-dependent mechanisms [112]. Notably, the function of Na_v_1.7 has been shown to be regulated by the MAPKs extracellular signal-related kinase 1/2 (ERK1/2). More specifically, the inhibition of phospho-ERK1/2, which phosphorylates Na_v_1.7, results in a depolarizing shift in the activation threshold. This impacts the resting membrane potential and firing properties of DRG neurons, leading to decreased neuronal excitability [113]. The role of Na_v_1.7 in mediating pain behaviors in the formalin test is underscored by observations in genetic models of Na_v_1.7 deficiency. In these models, nociceptive behaviors, such as the licking, flinching, and biting of the injected paw, are greatly reduced. Global Na_v_1.7 KO mice displayed dramatically reduced responses in both phases of the mouse formalin pain assay [92,105]. Positive behaviors in Phase I of the formalin response were reduced by approximately 80% in KOs versus control animals, whereas virtually no licking or lifting of the injected paw was observed during Phase II. A significant reduction in formalin-induced responses was also observed in both phases in the Na_v_1.7 nociceptor-specific KO mice (Na_v_1.7R^−/−^) [105]. Interestingly, in an tamoxifen-inducible KO mouse model, only a slight reduction in Phase I was observed, whereas Phase II displayed a delayed yet similar response in conditional KO mice compared to controls [91]. Accordingly, numerous Na_v_1.7 blockers, administered by various routes, have demonstrated efficacy in the formalin test [102,110,114,115].

## 6. Efficacy Studies and Role of Na_v_1.7 in Neuropathic Pain Models

Neuropathic pain is a pathological condition arising from injury to the somatosensory nervous system. It can be the result of nerve compression, channelopathy, autoimmune disease, infection, disease, or chemotherapy-induced neuropathy, among other causes. The most common types of human neuropathic pain include radiculopathy, postherpetic neuralgia, painful diabetic neuropathy, and neuropathic low back pain, highlighting the heterogenicity in pathophysiology, etiology, and clinical presentation of this condition [116].

Most of the neuropathic pain models used in preclinical studies, such as the spinal nerve ligation, spared nerve injury, and chronic constriction injury, are based on peripheral nerve injury, which promotes the degeneration of severed axons, Schwann cell activation, and the generation and release of chemokines, cytokines, and growth factors [47,50,57,117,118]. The activation of these signaling pathways sensitizes sensory nerve endings, induces inflammation, alters gene expression, promotes post-translational modification of proteins, and alters ion channel function, including that of Na_v_1.7 [119,120]. As a result, these models produce long-lasting pain in rodents, and the efficacy of compounds targeting Na_v_1.7 can be measured through their ability to reverse mechanical allodynia and/or thermal hyperalgesia.

### 6.1. CCI Model

Na_v_1.7 has been implicated in neural plasticity and the development of DRG neuronal sensitization and persistent pain in the CCI model. The thresholds for thermal and mechanical hyperalgesia are significantly reduced in CCI animals within 3 days post-surgery and remain altered for up to 28 days [49]. The development of hyperalgesia is accompanied by increased mRNA and protein expression of Na_v_1.7 in the DRG, suggesting that activation of peripheral nerve injury-induced nociceptive signals may increase Na_v_1.7 expression following sciatic nerve CCI [121,122]. Sirt1, a nicotinamide adenine dinucleotide (NAD)-dependent protein lysine deacetylase, and its downstream target, the microRNA miR-182, were shown to be downregulated in CCI rats [123]. Promoting Sirt1 activation with resveratrol increased paw withdrawal threshold, an effect associated with reduced Na_v_1.7 protein expression in the DRG. This finding further supports the critical role of Na_v_1.7 in pain processing following CCI surgery [123].

It has been shown that in DRG, sensory, and sympathetic neurons, Na_v_1.7 mediates the increased sensitivity to both the acetone and the von Frey test following CCI surgery, as Na_v_1.7^Advill^ and Na_v_1.7^Wnt1^ mice, which lack Na_v_1.7 in DRG and sensory sympathetic neurons, failed to develop hypersensitivity to the cold and mechanical tests after CCI surgery [35]. When Na_v_1.7 was specifically deleted from nociceptors using the Na_v_1.8 promoter (Na_v_1.7^Nav1.8^), mice subjected to CCI surgery did not develop acetone induced cold allodynia; however, mechanical allodynia was similar in Na_v_1.7^Nav1.8^ and control groups, suggesting that the development of mechanical and cold allodynia in this model requires distinct sets of neurons and signaling pathways. [35] Further validation of Na_v_1.7 as a promising target for analgesic drug development in neuropathic pain was provided in studies using an inducible DRG-specific Na_v_1.7 KO mouse strain driven by the Advillin-CreERT2 (Na_v_1.7^ADERT2^) promoter. In this model, tamoxifen-induced deletion of Na_v_1.7 effectively reversed mechanical allodynia induced by CCI surgery [35].

Consistent with these findings, Na_v_1.7 inhibitors have demonstrated significant analgesic properties following CCI surgery. For instance, intraperitoneal administration of a 3-arylindole-derived acylsulfonamide compound, identified as a potent and selective human Na_v_1.7 inhibitor, significantly reversed cold allodynia in mice [124]. Similarly, NeP1, a tocainide congener, dose-dependently reversed mechanical hyperalgesia in CCI rats [125]. Interestingly, analgesia can also be achieved through the indirect targeting of Na_v_1.7 by modulating its membrane expression via associated regulatory proteins such as the cytosolic phosphoprotein collapsin response mediator protein 2 (CRMP2) [126]. It has been demonstrated that either disruption of the motif responsible for the addition of a small ubiquitin-like modifier (SUMO) to CRMP2 or increasing CRMP2 deSUMOylation reduces the current density mediated by Na_v_1.7, but not Na_v_1.1 or Na_v_1.3, in neuronal cell lines and DRG neurons [127,128]. This effect was associated with decreased Na_v_1.7 surface expression, whereas total expression of the channel was not affected [127,129]. It has since been confirmed that inhibiting CRMP2 SUMOylation induces the recruitment of an endocytic protein complex to Na_v_1.7, leading to clathrin-mediated internalization of the channel, and resulting in decreased surface expression and current density [129]. Accordingly, oral administration of a compound that inhibits SUMO conjugation to CRMP2 significantly reversed mechanical allodynia in rats, further supporting the potential of both direct and indirect modulators of Na_v_1.7 activity as therapeutic strategies for neuropathic pain [126].

### 6.2. SNI Model

A time-dependent increase in Na_v_1.7 expression and membrane localization are observed in the L4-L6 DRG after SNI. This upregulation is observed 3 days after surgery and remains elevated even at 28 days post-surgery [51,130]. Moreover, these changes are not limited to an increase in Na_v_1.7 levels but are also associated with increased membrane localization of the channel in L4-5 DRG neurons, resulting in higher current densities within a subset of neuron subpopulation [21,130]. Pooling DRG neurons together is a common practice since the DRG contains only scarce amounts of tissue. However, immunofluorescent histochemistry and Western blot analysis of the L5 DRG of rats indicate a significant increase in Na_v_1.7 expression 14 and 21 days after SNI [52]. Of interest, some studies have actually demonstrated a down regulation of Na_v_1.7 mRNA expression in the DRG of both mice and rats following SNI surgery [56,131]. For example, separate analyses of L3, L4, and L5 DRG revealed that Na_v_1.7 mRNA levels were significantly lower in L3 DRG but remained unchanged in L4 and L5 [56]. The reasons for these discrepancies are not fully understood, but it should be pointed out that mRNA expression levels do not always correlate with amounts of protein and protein localization.

More insights into the role of Na_v_1.7 in mediating pain responses in this model were provided using tamoxifen-induced Na_v_1.7 adult KO mice (Na_v_1.7 cKO) [91]. In non-induced mice that underwent SNI surgery, hypersensitivity to cooling and mechanical stimuli were observed when assessed using acetone and von Frey filaments, respectively. However, the administration of tamoxifen to Na_v_1.7 cKO mice reversed cold allodynia but had no effect on mechanical allodynia, a finding not observed in the control animal. Similar effects in response to cold and mechanical stimuli were observed in a preventative paradigm, where tamoxifen was administered in mice prior to SNI surgery to induce Na_v_1.7 knockdown [91]. Nonetheless, suppressing the increase in Na_v_1.7 expression in the DRG following SNI surgery through microRNA miR-182 agomir treatment resulted in increased mechanical thresholds in rats [51]. Furthermore, reversal of mechanical allodynia in the SNI model has also been demonstrated following treatment with small molecules, single-domain antibodies, peptides, and others through different routes of administration, providing proof of concept that Na_v_1.7-selective blockade results in neuropathic pain relief [110,115,132,133].

Not only increased Na_v_1.7 expression but also modulation in its localization play a significant role in mediating pain in the SNI model. CRPM2 phosphorylation by cyclin-dependent kinase 5 (Cdk5) and conjugation to a small ubiquitin-like modifier (SUMOylated) have been shown to be increased in the spinal cord of rats following SNI surgery [134]. Inhibiting CRMP2 SUMOylation, which has been associated with Na_v_1.7 endocytosis, decreases in Na_v_1.7 currents and neuronal excitability and has been shown to reverse SNI-induced mechanical allodynia. In addition, pharmacological inhibition of CRMP2 phosphorylation, as well as blocking the domain responsible for the interaction between CRPM2 and Na_v_1.7, decreases Na_v_1.7 membrane localization and function in the DRG, leading to the reversal of mechanical allodynia following intrathecal administration in the SNI model of chronic neuropathic pain [128,134,135].

### 6.3. SNL Model

Anatomically, the spinal nerves of L4, L5, and L6 form the sciatic nerve and mediate the conduction of afferent signals. The skin of the foot is mainly innervated by L4 and L5 nerves, and the SNL model allows for a clear separation between injured and non-injured cell bodies since only the L5 spinal nerve is damaged [57]. It is hypothesized that the L4 nerve would be the one involved in the development of abnormal pain sensation in the SNL model since hyperalgesia and allodynia require an intact nerve to conduct the noxious information from the plantar skin of the hind paw to the spinal cord [136,137]. Accordingly, immunofluorescence studies demonstrated that Na_v_1.7 mRNA and protein levels are significantly higher in the ipsilateral L4 DRG of SNL operated animals, with a concomitant increase in the percentage of Na_v_1.7-positive neurons [121,131]. Additionally, *de novo* expression of Na_v_1.7 mRNA in putative proprioceptive neurons and increased Na_v_1.7-positive fibers in the gracile nucleus have also been observed, which may contribute to A-fiber spontaneous discharge and allodynia in the SNL model [138]. In contrast, a significant decrease in mRNA expression of most of the Na_v_s isoforms, including Na_v_1.7, has been reported in the injured L5 DRG in this model [56,121,131,138]. One possible explanation for this contradictory finding is that the complete transection of the L5 nerve in the SNL model prevents the conduction of peripheral afferent signals, resulting in a sharp decline in L5 DRG function, which is associated with reduced Na_v_1.7expression.

In Na_v_1.7 nociceptor-specific KO mice, following ligation of the L5 spinal nerve, animals developed mechanical allodynia to the same extent as their littermate controls [139]. Similarly, in the spinal nerve transection model of the fifth lumbar segment, deletion of Na_v_1.7 specifically in nociceptors using the Na_v_1.7^Nav1.8^ KO mice, or in the DRG using the Na_v_1.7^Advill^ KO mice, had no impact on the development of cold and mechanical allodynia [93]. However, when Na_v_1.7 was deleted from sensory and sympathetic neurons using the Wnt1 promoter in Na_v_1.7^Wnt1^, no cold or mechanical allodynia was developed, suggesting that Na_v_1.7 expression in peripheral sympathetic neurons is essential for pain transmission in the spinal nerve transection model [93,94]. Interestingly, an attempt to generate a humanized Na_v_1.7 rat expressing a chimeric Na_v_1.7 protein (HOM-KI) had an unexpected outcome. The human insert was spliced out during transcription, resulting in a premature stop codon. Consequentially, Na_v_1.7 protein was not detected in the nervous system, including the brainstem, DRG, and sciatic nerve, although it was still present in the olfactory sensory neurons. As a result, in these rats, tactile and cold allodynia were abolished [140].

As in other neuropathic pain models, pharmacological inhibition of CRMP2 SUMOylation leads to decreased Na_v_1.7 currents through clathrin-mediated internalization of Na_v_1.7, thereby restoring mechanical sensitivity [128]. Whereas in the SNI model, this effect was only observed by intrathecal injection, in the SNL, oral administration was able to induce reversal of mechanical allodynia [128]. Similarly, oral administration of numerous bioavailable compounds with optimal Na_v_1.7 selectivity, or those that selectively stabilize ion channels in their slow-inactivated state, were shown to reverse thermal hyperalgesia and tactile allodynia in the rat SNL model [141,142,143].

### 6.4. SCI Models

The role of Na_v_1.7 in SCI has been studied in a partial spinal transection injury model, where a defined lesion is unilaterally performed in the right dorsal horn at the T13 level [144]. Mechanical pain was observed in the ipsilateral paws of SCI mice within 3 days post-surgery. This was accompanied by a significant increase in Na_v_1.7 expression in the L4-6 DRG [144]. Moreover, ectopic expression of Na_v_1.7 was detected in spinal dorse horn neurons located in both the superficial and deep layers surrounding the damaged spinal area. Not surprisingly, the intraperitoneal injection of Na_v_1.7 blockers, such as PF-05089771 and GNE-0439, significantly alleviated mechanical allodynia, further supporting the role of Na_v_1.7 upregulation in the development of pain in this SCI model [144].

### 6.5. CIPN Models

The evidence supports a crucial role for Na_v_1.7 in mediating the development of sensory neuropathy following paclitaxel treatment in both humans and rodents. Treatment with this anti-neoplastic agent increases Na_v_1.7 protein and mRNA expression in sensory and DRG neurons, which correlates with enhanced excitability and onset of mechanical allodynia [145,146,147]. Paclitaxel has been shown to induce ERK1/2 phosphorylation, which, in turn, colocalizes with Na_v_1.7 in DRG neurons and phosphorylates multiple sites of Na_v_1.7, thus modulating the Na_v_1.7 channel gating properties and further enhancing DRG neuronal excitability [148]. These findings have been further supported by in vitro studies demonstrating that low concentrations of paclitaxel modulate intra-axonal trafficking of Na_v_1.7-containing vesicles, increasing Na_v_1.7 membrane localization and distribution at the surface of axon terminals [149]. Conversely, epigenetic strategies to knockdown Na_v_1.7 expression or an injection of Na_v_1.7 antibodies results in reversal of the neuropathic pain phenotype in mice with CIPN [107,146].

The analgesic potential of numerous compounds targeting Na_v_1.7 has been shown in paclitaxel-induced neuropathy. DA-0218, a small-molecule Na_v_1.7 inhibitor discovered using a computer-aided drug design, transiently but completely reversed paclitaxel-induced mechanical allodynia for several hours when administered intrathecally [150]. Interestingly, no effect on allodynia was observed when DA-0218 was administered intraperitoneally, even though the same dose used was effective in reducing formalin-induced inflammatory pain [150]. Notably, the same study reported that intrathecal injection of PF-05089771 (10 nmol and 30 nmol) failed to produce any antiallodynic effect in the paclitaxel model [150]. More recently, a study showed that Na_v_1.7-targeted gene therapy reversed mechanical and cold allodynia in the paclitaxel-induced peripheral neuropathy model [135]. This was achieved with a genetically encoded CRMP2 regulatory sequence peptide that was shown to reduce Na_v_1.7 currents and trafficking, providing proof-of-concept for a novel strategy targeting Na_v_1.7 for the treatment of chronic pain [135].

Oxaliplatin, a platinum-based antineoplastic medication, is also extensively used in cancer therapy [151]. Nonetheless, painful neuropathy is a severe adverse effect of oxaliplatin treatment that significantly compromises quality of life, ultimately leading to the discontinuation of chemotherapy in patients [152]. Although evidence suggests that genetic variants in SCN9A are associated with distinct pain perception in oxaliplatin-treated cancer patients, the role of Na_v_1.7 mediating oxaliplatin-induced pain remains controversial [35,153]. For instance, mice with specific deletion of Na_v_1.7 in DRG and sympathetic neurons developed mechanical and cold allodynia following oxaliplatin treatment, similar to their littermate controls [35]. This suggests that unlike other acute, inflammatory, and neuropathic pain behaviors, Na_v_1.7 expression within the PNS is not required for oxaliplatin-induced pain [35]. On the other hand, increased Na_v_1.7 mRNA and protein levels have been observed in the DRG of oxaliplatin-treated rats within 7 days [154]. This is associated with bilateral allodynia as no differences between the left and right paw withdrawal thresholds were generally observed [154,155]. Moreover, intrathecal administration of a selective Na_v_1.7 channel blocker, ProTx II, significantly attenuated oxaliplatin-induced mechanical allodynia [154]. Furthermore, as previously reported in other pain models, inhibition of CRMP2 SUMO conjugation, which results in reduced membrane localization of Na_v_1.7 and Na_v_1.7-mediated sodium currents, significantly reversed oxaliplatin-induced mechanical allodynia for up to 3 h post-treatment [155]. Interestingly, treatment with a CRMP2 SUMO conjugation inhibitor concomitantly with oxaliplatin successfully prevented the development of mechanical allodynia in the platinum complex agent-treated mice, potentially suggesting a role for this compound as an adjuvant therapy in the clinic [155].

## 7. Na_v_1.7 in the Plantar Incision Model of Post Operative Pain

Mechanical and thermal pain threshold decreases significantly within 2 h of plantar incision surgery. This hyperalgesia has been shown to be mediated by the production of inflammatory cytokines, including neural growth factor and brain-derived growth factor, as well as an increase in the spontaneous activity of DRG neurons [156]. Of interest, increased mRNA and protein expression of Na_v_1.7 in the L4–L6 DRG within 72 h post-surgery has been reported [157,158,159]. This upregulation is mediated through nerve growth factor, tropomyosin receptor kinase A (TrkA), and serum glucocorticoid-regulated kinase 1 (SGK1)-dependent signaling pathways [159]. Clinical validation of the role of Na_v_1.7 in postoperative pain was provided by studies showing that that genetic polymorphisms in SCN9A affect postoperative pain sensitivity in surgical patients [160]. Preclinical studies further highlight the role of Na_v_1.7 in mediating postoperative pain. Pre-treatment of rats with SCN9A siRNA lentivirus to knockdown the increase in Na_v_1.7 expression in the DRG significantly alleviates pain hypersensitivity after plantar incision [157]. Of note, in the tamoxifen-induced Na_v_1.7 adult KO mice, a reversal of thermal hypersensitivity, but not of mechanical hypersensitivity, was observed in the incision model of postoperative pain [91]. Similarly, ST-2530, a potent and selective Na_v_1.7 inhibitor developed through rational modification of the guanidinium toxin saxitoxin, effectively reversed thermal hypersensitivity induced by plantar incision [161].

## 8. Role of Na_v_1.7 in OA Models

One of the main clinical symptoms of OA is pain. In joint tissues, specialized peripheral sensory neurons are abundant and contribute to pain in OA. These neurons express unique repertoires of Na_v_s, including Na_v_1.7 [162]. Not surprisingly, gain-of-function mutations in Na_v_1.7 increase pain sensitivity in some patients with OA [163]. Moreover, Na_v_1.7 mRNA has been shown to be upregulated in cartilage from OA patients when compared with non-arthritic cartilage [83]. Chronic inflammatory joint pain has been shown to be associated with a significant increase in the number of Na_v_1.7 positive neurons of L3–L5 DRG [84]. The relative contribution of Na_v_1.7 expressed in DRG and chondrocytes to OA disease progression and pain was further investigated by generating mice with regional deletion of Na_v_1.7 in DRG neurons, chondrocytes, or both [83]. Histological analysis revealed that Na_v_1.7 deletion in both DRG neurons and chondrocytes substantially attenuated cartilage loss in surgical and chemical OA models. This was accompanied by reduced mechanical allodynia and improved open-field movement activity [83]. Similar results were observed in mice with specific knockdown of Na_v_1.7 in chondrocytes. Interestingly, deletion of Na_v_1.7 in DRG neurons reduced pain without affecting structural abnormalities such as cartilage destruction, osteophyte formation, or other pathological scores in a surgically induced model of OA [83]. The role of DRG-expressed Na_v_1.7 in OA pain was further supported by the reversal of the evoked neuronal response to noxious mechanical stimulation following an intrathecal injection of ProTx II in the MIA model [164]. Taken together, these findings indicate that Na_v_1.7 channels expressed in chondrocytes modulate OA disease progression and the associated pain behavior, whereas Na_v_1.7 expressed in DRG neurons contributes to OA-associated pain without affecting chondrocyte biology [83].

The effectiveness of Na_v_1.7 blockade in preventing pain associated with OA and disease progression has been demonstrated. Systemic administration of PF-04856264 through oral gavage markedly slowed OA progression, as demonstrated by decreased cartilage loss and increased movement in the open field test, as well as the mechanical hind paw withdrawal threshold [83]. In addition, intra-articular injection of PF-04856264 protected the structure of articular cartilage and maintained proteoglycan content in the cartilage in an OA model [83]. Similarly, indirect inhibition of Na_v_1.7 activity by modulating its membrane expression with a compound that prevents SUMOylation of CRMP2 dose-dependently reduced MIA-induced mechanical and cold allodynia [165].

## 9. Challenges and Future Direction

As summarized in Table 1, studies across numerous pre-clinical models have validated Na_v_1.7 as a promising target for therapeutic intervention and provided valuable insights into its role in pain transmission and underlying biology (Table 1). However, despite robust pre-clinical evidence demonstrating the efficacy of Na_v_1.7 blockers in reducing pain, its clinical translation has yielded rather disappointing results. This emphasizes the need for more innovative and refined approaches, as discussed by Eagles and cols [29].

Patients with chronic pain exhibit significant heterogeneity in pathophysiology, etiology, and clinical presentation. This is in contrast with the majority of pain models employed in pre-clinical studies, which fail to capture the heterogenous disease mechanisms observed in patients [166,167]. This poor correlation between preclinical test subjects and the clinical patient population likely contributes to the translational challenges associated with the development of novel analgesics, i.e., not only those targeting Na_v_1.7 but also a broader range of analgesic compounds. For instance, most preclinical studies are performed in young, healthy, male rodents of a genetically similar background. In contrast, neuropathic pain in clinical settings is most commonly found in middle-aged or elderly patients with heterogeneous genetic profiles [168]. Notably, a study comparing paclitaxel-induced neuropathy in CD1 mice of different ages found that juvenile and aged mice exhibited more severe mechanical allodynia and thermal hyperalgesia compared to young adult mice [169]. Moreover, different mouse strains showed variable sensitivity to pain in different behavioral assays, further highlighting the limitations of using a single-rodent model/mouse strain to test candidate compounds in preclinical studies [170]. Sex differences in pain are also increasingly recognized as critical factors in understanding chronicity, severity, and response to analgesics. Given that a significant proportion of chronic pain patients are female, there is a growing interest in examining these differences, which may have profound implications for the translational efficacy of new therapies [171,172]. Finally, differences in pain processing and assessment between rodents and humans present challenges for extrapolating preclinical findings. To mitigate this issue, the use of larger animal models and human tissues in these preclinical studies is recommended in order to bridge this knowledge gap, as reviewed and discussed by Sadler and colleagues [173]. Taken together, it has increasingly been accepted that a broader range of validated and long-lasting pain assays is essential to fully determine the efficacy of a therapeutic drug. Incorporating operant and non-invasive protocols for assessing analgesia in pre-clinical settings may also improve the translation of animal-based observations into effective human therapeutics.

A key element in drug discovery is delineating the relationship between the in vitro potency, the pharmacokinetic properties of a compound, its drug exposure at site of action, target engagement, the desired pharmacological response, and overall efficacy, all while monitoring for signs of toxicity and performing safety assessments [174,175,176]. For drugs targeting Na_v_1.7, an additional requirement may include the ability to cross the blood–brain barrier and the blood–nerve barrier as Na_v_1.7 is expressed in the central terminal of DRG neurons in the dorsal horn, which lies behind the blood–spinal cord barrier [177]. As such, targeting Na_v_1.7 activity in the dorsal horn might be necessary to effectively inhibit pain signaling. These considerations are particularly important considering the failure in translating the pre-clinical success of Na_v_1.7 into clinical efficacy. To date, despite demonstrating effectiveness in some animal models, the selective small-molecule Na_v_1.7 inhibitors tested in clinical trials have failed to meet the defined efficacy criteria. Uncertainty remains around the exact level of the Na_v_1.7 blockade necessary to achieve analgesia [27]. A further look into its properties identified that it was unable to achieve sufficient coverage of the Na_v_1.7 IC50 to effectively engage the target in vivo in both rodents and non-human primates [111,178].

Na_v_1.7 selective inhibitors may impact the autonomic nervous system owing to the expression of Na_v_1.7 in sympathetic tissues. mRNA for Na_v_1.7 channel has been reported in 82% of the sympathetic neurons isolated from the stellate ganglion [179,180]. A previous report described the presence of Na_v_1.7 mRNA in the postganglionic parasympathetic nerves regulating human and guinea-pig airway smooth muscle, while no Na_v_1.7 mRNA was observed in the same nerves in mice [181]. Consequently, Na_v_1.7 blockers could inhibit the sympathetic nerve-mediated adrenergic contraction of blood vessels and abolish parasympathetic nerve-mediated cholinergic contractions of the airway smooth muscle, suggesting that Na_v_1.7 blockers could have profound effects on the sympathetic and parasympathetic systems [180,181]. This is corroborated by observations that patients with hereditary erythromelalgia experience flushing, which may be related to altered sympathetic neurons activation and vasodilation [182]. In addition, hypotensive effects following intravenous administration of Na_v_1.7 inhibitors have been reported in rodents, non-human primates, and humans [183,184,185]. Telemetry data showed that ST-2560 (a selective inhibitor of Na_v_1.7 with IC50 of 39 nM) induced a dose-dependent reduction in systolic and diastolic blood pressure in conscious, free-moving cynomolgus monkeys. This effect was transient, with blood pressure gradually returning to baseline levels within 5 hours post-dosing [185]. Similarly, in the clinical testing of MK-2075, another small-molecule selective Na_v_1.7 inhibitor, orthostatic hypotension linked to Na_v_1.7-dependent cardiac autonomic dysfunction was observed in patients administered with either 30 mg by intravenous infusion over 8 h or 8 mg over 2 h [186]. These findings highlight the necessity of characterizing the effects of potential Na_v_1.7-targeting candidates on the autonomic nervous system and cardiovascular endpoints. This is particularly important for molecules capable of achieving high levels of target engagement as acute inhibition of Na_v_1.7 could also lead to autonomic side effects, including reduced heart rate variability and impaired spontaneous baroreceptor sensitivity.

Side effects due to off-target binding to other Na_v_ subtypes are another major concern when developing compounds targeting Na_v_1.7. One possible solution is the generation of antibodies against unique epitopes of Na_v_1.7. Although promising, this strategy faces several challenges that must be addressed in order to succeed. Na_v_s are considered difficult targets for antibody development due to (i) the high degree of similarity in amino acid sequence and tertiary structures among Na_v_1.x isoforms; (ii) their numerous transmembrane segments; (iii) the short extracellular domains; and (iv) the different steric conformations they adopt depending on their kinetic state (i.e., closed, active/open, inactivated) [187]. Compared to small molecules, antibodies have the significant advantage of recognizing highly specific amino acid sequences. When this sequence is unique to a particular isoform, antibodies can achieve exceptional target specificity. However, while some monoclonal antibodies against Na_v_1.7 have been developed (e.g., SVmab), they lack functional activity, despite successfully binding to the channel. Better success was achieved by Martina and collaborators, who developed a novel antigen design and presentation strategy, resulting in the generation of a single-domain antibody (V_H_H DI-D) with analgesic effect in animal pain models, including the OD1 and formalin pain models in mice and the Hargraves and spare nerve injury pain models in rats [110]. The epitope targeted by V_H_H DI-D is located on the third extracellular loop of domain DIII and is unique to Na_v_1.7. Unlike small molecules, the mode of action of V_H_H DI-D does not involve preventing the movement of the voltage sensor upon changes in membrane voltage (as spider toxins do) or obstructing the conductive pathway (as marine toxins do). Instead, it slows the deactivation process of the channel, thus decreasing the firing capability of sensory neurons. This mechanism is probably a key factor in its analgesic efficacy in rat and mouse preclinical pain models [110].

Another promising strategy consists of developing molecules that specifically modulate Na_v_1.7 activity by interacting with the intracellular proteins regulating its membrane trafficking. One such molecule was developed by the company Regulonix and called compound 194. Derived from a class of benzoyl piperidyl benzimidazole, compound 194 inhibits the addition of SUMO onto the Na_v_1.7-interacting cytosolic CRMP2. This mechanism effectively blocks Na_v_1.7 function by preventing its trafficking to the cell surface [128]. As discussed previously in this review, compound 194 displayed robust antinociceptive properties in multiple preclinical models of acute and chronic pain when administered not only intrathecally but also via the oral route, which is very relevant when considering its potential clinical translation [124,125,126,127,128,134,135].

Agents targeting other ion channels are subject to active drug discovery and development efforts. A significant breakthrough was recently achieved when the U.S. Food and Drug Administration approved suzetrigine (VX-548), a first-in-class Na_v_1.8 inhibitor, for the treatment of moderate-to-severe acute pain [188]. Interest in Na_v_1.9 modulators has grown since the discovery that mutations in its gene are associated with painful neuropathies [189]. ANP-230, which inhibits Na_v_1.7, Na_v_1.8, and Na_v_1.9 with similar potencies, is currently undergoing clinical trials in Japan (jRCT2061200046). Additionally, calcium, potassium, and acid-sensing ion channels are also being targeted in chronic pain control [190,191,192]. Considering their role in conveying thermal, mechanical, and chemical stimuli signals in afferent sensory neurons, it is not surprising that TRP channels are also being targeted for pain relief. Clinical trials are currently underway for agents against TRPA-1, TRPV-1, TRPV-3, and TRPM-8 [193,194,195]. Nevertheless, adverse effects and, in some cases, lack of therapeutic efficacy in clinical trials hamper the development of compounds targeting TRP channels, highlighting the similar challenges faced in the discovery of Na_v_1.7 inhibitors, as previously discussed in this review.

## 10. Conclusions

In summary, the available genetic and pharmacological evidence strongly supports the development of new molecules targeting Na_v_1.7 for various—though not all—types of pain in which this channel plays a role. However, there is a pressing need for a robust translational workflow that incorporates in vivo characterization across multiple models, reflecting the heterogenicity of pain presentation observed in the clinic. In addition, it is increasingly evident that achieving high specificity for Na_v_1.7 alone is insufficient for the development of a successful analgesic. Novel mechanisms of action must be explored, and pharmacokinetic, pharmacodynamic, and toxicity profiles should be carefully evaluated early in the drug development process. This comprehensive approach is essential to overcoming the challenges of translating preclinical findings into clinically effective therapies targeting this channel for pain management in humans.

## Figures and Tables

**Table 1:** Summary of test compounds and genetic models, demonstrating the role of Nav1.7 in pain models.

Pain Model	Test Compound	Class	Genetic Model	Reference
*Inflammatory*				
CFA	-	-	Na_v_1.7 cKO, Na_v_1.7 gKO Na_v_1.7R^−/−^	[91,92,105]
	Compound 52	Aminotriazine	-	[102]
	GX-201, GX-201, GX-585	Acylsulfonamide	-	[91,111]
	NAN-190	5-HT_1A_ antagonist	-	[108]
	GpTx-1-71	Venom peptide	-	[109]
	V_H_H DI-D	SdAb		[110]
Carrageenan	-	-	Na_v_1.7R^−/−^	[105]
	ZFP-KRAB	Epigenetic platform	-	[146]
Formalin	-	-	Na_v_1.7 gKO, Na_v_1.7R^−/−^	[92,105]
	Compound 52	Aminotriazine	-	[102]
	V_H_H DI-D	SdAb	-	[110]
	μ-conotoxin KIIIA analogues	Venom peptide	-	[114]
	QLS-278	Acylsulfonamide derivative	-	[115]
*Neuropathic*				
CCI	-	-	Na_v_1.7^Advill^, Na_v_1.7^Nav1.8^, Na_v_1.7^Wnt1^	[35]
	Compound 29	Acylsulfonamide derivative	-	[124]
	NeP1	Tocainide congener	-	[125]
	Compound 194	CRMP2 SUMOylation inhibitor	-	[126]
SNI	-	-	Na_v_1.7 cKO	[91]
	miR-182 agomir	MicroRNA	-	[51]
	V_H_H DI-D	SdAb	-	[110]
	QLS-278	Acylsulfonamide derivative	-	[115]
	μ-TRTX-Hhn1b	Venom peptide	-	[132]
	Compound 3 g	3-Hydroxyindole backbone	-	[133]
	Compound 194	CRMP2 SUMOylation inhibitor	-	[128]
	Myr-TAT-Na_v_1.7-CRS	CRMP2/Na_v_1.7 interaction inhibitor	-	[135]
SNL	-	-	Na_v_1.7^Wnt1^, Na_v_1.7^Advill^ (no effect), nociceptor-specific Nav1.7 KO, HOM-KI rats	[93,139,140]
	Compound 194	CRMP2 SUMOylation inhibitor	-	[128]
	Z123212	Piperazine derivative	-	[141]
	Compound 33	Quinoline amide	-	[142]
	Compound 9	Aminocyclohexene analogue	-	[143]
SCI	PF-05089771	Arylsulfonamide	-	[144]
	GNE-0439	VSD4 domain	-	[144]
CIPN (Paclitaxel)	ZFP-KRAB, KRAB-dCas9	Epigenetic platform	-	[146]
	DA-0218	Propanamide, methoxyphenyl, benzyl indolyl derivative	-	[150]
	Myr-TAT-Na_v_1.7-CRS	CRMP2/Na_v_1.7 interaction inhibitor	-	[135]
CIPN (Oxaliplatin)	-	-	Na_v_1.7^Advill^, Na_v_1.7^Wnt1^ (no effect)	[35]
	ProTx II	Venom peptide	-	[154]
	Compound 194	CRMP2 SUMOylation inhibitor	-	[155]
*Post operative*				
Plantar incision	-	-	Na_v_1.7 cKO (effect on thermal but not mechanical response)	[91]
	SCN9A-RNAi-LV	siRNA lentivirus	-	[157]
	ST-2530	Saxitoxin analog	-	[161]
*Osteoarthritis*				
MIA	-	-	Nav1.7^DRG;chondrocyte^	[83]
	PF-04756264	Arylsulfonamide	-	[83]
	ProTx II	Venom peptide	-	[164]
	Compound 194	CRMP2 SUMOylation inhibitor	-	[165]

HOM-KI rats (humanized Na_v_1.7 rat expressing a chimeric Na_v_1.7 protein); Na_v_1.7 cKO (tamoxifen-inducible Nav1.7 knockout); Na_v_1.7 gKO (global Na_v_1.7 knockout mouse); Na_v_1.7^Advill^ (all sensory neuron Nav1.7 knockout); Na_v_1.7^DRG;chondrocyte^ (DRG and chondrocyte Na_v_1.7 knockout); Na_v_1.7^Nav1.8^ (Na_v_1.8 positive nociceptor Na_v_1.7 knockout); Na_v_1.7R^−/−^ (tissue-restricted Na_v_1.7 knockout); Na_v_1.7^Wnt1^ (sensory and sympathetic neurons Na_v_1.7 knockout); SdAb (single-domain antibody).

## Data Availability

No new data were created or analyzed in this study. Data sharing is not applicable to this article.

## Abbreviations

CCI	Chronic constriction injury
Cdk5	Cyclin-dependent kinase 5
CFA	Complete Freund’s adjuvant
CIP	Congenital insensitivity to pain
CIPN	Chemotherapy-induced peripheral neuropathy
CNS	Central nervous system
CRMP2	Collapsin response mediator protein 2
DRG	Dorsal root ganglia
EM	Erythromelalgia
ERK1/2	Extracellular signal-related kinase 1/2
MIA	Monoiodoacetate arthritis
MAPK	Mitogen-activated protein kinase
NAD	Nicotinamide adenine dinucleotide
Na_v_1.7	Voltage-gated sodium channel subtype 1.7
Na_v_s	Voltage-gated sodium channels
OA	Osteoarthritis
OD1	First α toxin from *Odonthobuthus doriae* scorpion
PEPD	Paroxysmal extreme pain disorder
PNS	Peripheral nervous system
SCI	Spinal cord injury
SGK1	Serum glucocorticoid-regulated kinase 1
SNI	Spared nerve injury
SNL	Spinal nerve ligation
SUMO	Small ubiquitin-like modifier
TrkA	Tropomyosin receptor kinase A
TRP	Transient receptor potential
VSD	Voltage-sensing domain
